# The Efficacy, the Treatment Response and the Aquaretic Effects of a Three-Year Tolvaptan Regimen in Polycystic Kidney Disease Patients

**DOI:** 10.3390/clinpract13050092

**Published:** 2023-08-24

**Authors:** Vasiliki Gkika, Michaela Louka, Mihail Tsagkatakis, George Tsirpanlis

**Affiliations:** 1 Department of Nephrology, General Hospital of Athens “G. Gennimatas”, 11527 Athens, Greece; vasilikigkika91@gmail.com (V.G.); mixaellalouka@gmail.com (M.L.); 2 Department of Magnetic Resonance, Bioiatriki, 17563 Nea Smirni, Greece

**Keywords:** ADPKD, tolvaptan, prediction for ESKD, aquaretic adverse effects

## Abstract

Tolvaptan, a selective vasopressin V2 receptor antagonist, is the first and only approved specific treatment for Autosomal-Dominant Polycystic Kidney Disease (ADPKD), and is used in current clinical practice. Real clinical data are missing. In this retrospective study, 41 ADPKD patients received tolvaptan for 3 years, from 2018 to 2021. Total kidney volume (TKV) was measured using Magnetic Resonance Imaging, at initiation and at the end of the treatment period. A complete biochemistry/hematology profile and a 24 h urine volume collection were performed monthly for the first 18 months and every 3 months thereafter. At the end of the treatment period, the median (IQR) estimated Glomerular Filtration Rate (e-GFR) was 5.3 (−1.3, 8.7) mL/min higher than the expected e-GFR decline without treatment, while the prediction for End Stage Chronic Kidney Disease (ESKD) had been prolonged by 1 (0, 2) year. Total Kidney Volume did not change significantly (2250 (1357) mL at 3 years of treatment vs. 2180 (1091) mL expected without treatment, *p* = 0.48). Younger patients with a relatively preserved e-GFR, lower hypertension burden, better familiar renal prognosis and more severe imaging data showed better outcomes. The aquaretic adverse effects of tolvaptan did not affect renal function and electrolyte balance in 51 patients, in a follow-up period of 18 months. Consequently, tolvaptan seems to be effective in preventing progression of ADPKD when administered in a timely manner in patients with better familiar renal history, shorter hypertension duration and worse imaging profile. Increased diuresis does not affect treatment efficacy.

## 1. Introduction

Autosomal-Dominant Polycystic Kidney Disease (ADPKD) is the most common monogenic inherited kidney disease worldwide (at least 10:10,000 individuals) [1]. Tolvaptan, a vasopressin V2 receptor antagonist, was approved in 2016 as the first specific treatment for the disease. Approval was based on the results of TEMPO 3:4 and REPRISE studies [2,3]. Real long-term clinical experience with this drug in ADPKD patients has not been published and the most common adverse effect of the treatment, namely, the aquaretic effect (i.e., the massive increase in urine volume), has not been studied in daily practice.

The aim of the present retrospective study was to assess, in real clinical conditions, (1) the tolvaptan effectiveness in terms of kidney volume, renal function and renal prognosis in a 3-year treatment regimen; (2) the factors that influenced the response to treatment and (3) the aquaretic adverse effects and their possible burden on renal function and electrolyte balance.

## 2. Materials and Methods

### 2.1. Study Population

Forty-one patients diagnosed with ADPKD (echographically and by genetic testing in those with a clear and unclear family history, respectively) were included in the study. All patients received tolvaptan for at least 3 years, from 2018 to 2021. Patients that withdrew treatment for any cause were not included in the study (2 patients withdrew treatment in the first 6 months due to hepatotoxicity and 1 patient because of the aquaretic adverse effects). Eligibility criteria for tolvaptan prescription in ADPKD patients were age 18–55 years old, an estimated Glomerular Filtration Rate (e-GFR) >25 mL/min and a Mayo Clinic Imagining Category (MCIC) 1C, 1D or 1E [4]. Total kidney volume (TKV) was measured using Magnetic Resonance Imaging (MRI) at initiation and after three years of treatment. A complete biochemistry/hematology profile, including urine osmolarity and a 24 h urine volume collection, were performed monthly for the first 18 months and every 3 months thereafter. In order to reduce the risk of significant liver injury, we followed all the required safety measures. In addition, we concurrently monitored for symptoms of liver injury such as anorexia, nausea, vomiting, right upper abdominal discomfort, rash and jaundice. In cases where a patient showed abnormal ALT, AST or BT levels (ALT or AST > 3 times upper limit normal (ULN) and BT > 2 times ULN; International Normalized Ratio [INR] > 1.5 or with persistent symptoms of hepatic injury), tolvaptan was discontinued. Fifty-one patients, treated with the drug for 18 consecutive months, were included in the part of the study focusing on the aquaretic diverse effects of tolvaptan. The need for informed consent was waived because of the retrospective design of the study.

### 2.2. Clinical and Laboratory Assessment

The MCIC was determined for all patients before treatment and 3 years post-treatment (https://www.mayo.edu/research/documents/pkd-center-adpkd-classification/doc-20094754). The expected e-GFR decline at 3 years without treatment was calculated using two different methods: (1) Mayo Clinic Formula [5]; (2) taking into consideration the decline rate in renal function by Chronic Kidney Disease (CKD) stage observed in the placebo group of TEMPO 3:4 trial (−2.55 mL/min/year for CKD stage 1, −3.90 mL/min/year for CKD stage 2 and −5.6 mL/min/year for CKD stages 3 and 4) [6]. The expected TKV increment without treatment at 3 years was calculated (5.3% increment per year) [7] and compared to the TKV measured after 3 years of tolvaptan treatment. Finally, the expected renal prognosis (prediction of End Stage CKD as defined by an e-GFR of 10 mL/min) at the time of treatment initiation was calculated using the aforementioned Mayo Clinic formula and compared to the renal prognosis calculated with the same formula, based on real patients’ data found after 3 years of tolvaptan treatment [5].

### 2.3. Statistical Analysis

Descriptive statistics are reported as mean ± standard deviation in normally distributed continuous variables, as median and interquartile range in skewed continuous variables and as frequency percentage for binary data. The normal distribution of all continuous variables was tested with the parametric Shapiro–Wilk normality test. Differences between sex were determined by an independent sample *t*-test or a nonparametric Mann–Whitney test, in normally and skewed continuous variables, respectively, and the chi-square followed by a Fisher’s exact test for categorical variables (frequency distributions). The expected and measured e-GFR, ESKD prediction and TKV were compared with the use of paired *t*-test or the Wilcoxon signed-rank test. Multivariable linear regression was utilized in the analysis of the above-mentioned differences in e-GFR and ESKD prediction. The regression models included age, sex, age at ADPKD diagnosis, presence of hypertension (HTN) and age at HTN onset, age at ESKD of the affected parent, TKV and MCIC as covariates. Results were expressed as beta coefficients (β) and 95% confidence intervals (CI). In addition, we performed longitudinal analysis to detect possible aquaretic effects associated with tolvaptan treatment. The Stata/MP 14.0 software was applied to analyze all data, and the significance level was set at 0.05 in all cases.

## 3. Results

The baseline patient characteristics before initiation of tolvaptan treatment are shown in Table 1. The dose of tolvaptan was adjusted according to the urine osmolarity (<200 mOsm/Kg). In the third year of treatment, 15, 13 and 13 patients were treated with tolvaptan 90/30, 60/30 and 45/15 mg/day, respectively.

### 3.1. The Tolvaptan Effectiveness on Kidney Volume, Renal Function and Renal Prognosis

The expected TKV and e-GFR (calculated with two different methods (1,2) as described earlier) at 3 years without treatment, as well as ESKD prediction at the initiation of tolvaptan treatment versus the aforementioned parameters measured after 3 years of tolvaptan treatment, are shown in Table 2.

The median (IQR) difference between e-GFR measured after three years of tolvaptan treatment and the expected e-GFR without treatment was 5.3 (−1.3, 8.7) mL/min. The median (IQR) ESKD prediction without treatment calculated prior to treatment initiation, had been prolonged by 1 (0, 2) year after 3 years of tolvaptan treatment. In univariate analysis, the slower than expected e-GFR decline and the prolongation in time of ESKD prediction after 3 years of tolvaptan treatment were associated with the patient’s e-GFR at treatment initiation (β = 0.16, *p* < 0.02, and β = 0.04, *p* < 0.01, respectively) and with the age of ESKD onset in the affected parent (β = 0.35, *p* < 0.047 and β = 0.1, *p* < 0.021, respectively).

### 3.2. The Factors That Influenced the Response to Treatment

**Figure 1:**
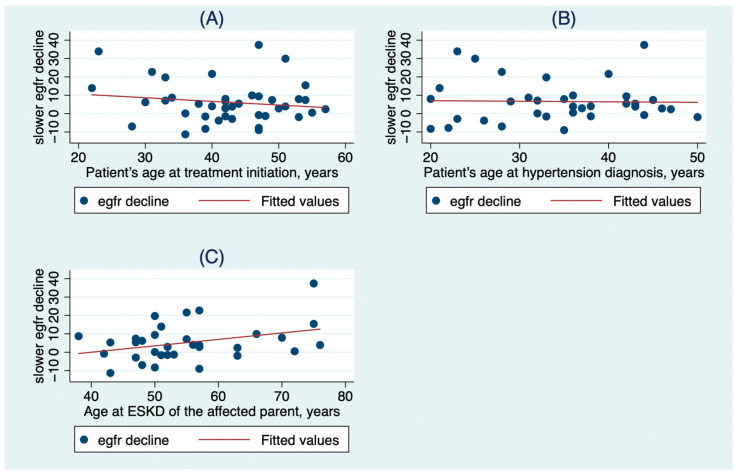
The association of slower e-GFR decline after 3 years of with (**A**) the patient’s age at treatment initiation, (**B**) the patient’s age at hypertension diagnosis, and (**C**) the age at ESKD of the affected parent.

In multivariable analysis, factors with a significant impact on the slower than expected e-GFR decline after 3 years of tolvaptan treatment were the patient’s age at treatment initiation (β = −0.9, 95% CI: −1.52 to 0.29, *p* = 0.006), the patient’s age at hypertension diagnosis (β = 0.88, 95% CI: 0.25 to 1.52, *p* = 0.008) and the age at which the affected parent had developed ESKD (β = 0.57, 95% CI: 0.16 to 0.98, *p* = 0.008) (Table 3, Figure 1).

**Figure 2:**
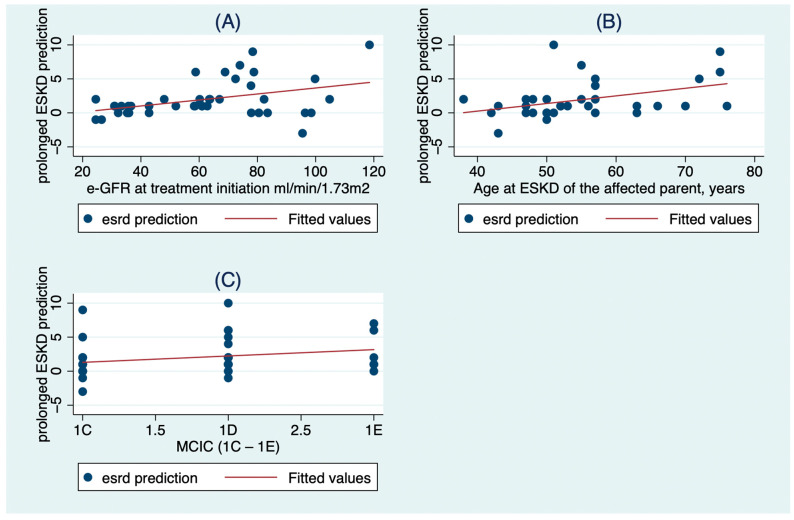
The association of prolonged ESKD prediction after 3 years of tolvaptan with (**A**) the e-GFR at treatment initiation, (**B**) the age at ESKD of the affected parent, and (**C**) the Mayo Clinic Imaging Category (MCIC).

Table 4 and Figure 2 show the factors that were associated with the prolongation in ESKD prediction time after 3 years of tolvaptan treatment. The factors were the e-GFR at treatment initiation (β = 0.6, 95% CI:0.03 to 0.1, *p* = 0.001), the age at ESKD of the affected parent (β = 0.13, 95% CI:0.03 to 0.23, *p* = 0.013) and the Mayo Clinic Imaging Category (β = 1.93, 95% CI: 0.46 to 3.39, *p* = 0.013).

### 3.3. The Aquaretic Adverse Effects

In total, 51 ADPKD patients, 19 females and 32 males, mean age of (SD) 41.9 (8.9) years old, treated with the drug for at least 18 months, were included in the part of the study investigating the aquaretic adverse effects of tolvaptan. Of these, 20 patients (39%) were in MCIC 1C, 22 (43%) in 1D and 9 (18%) in 1E, while 9 (18%) were at CKD stage 1, 16 (31%) at stage 2, 6 (12%) at 3α, 13 (25%) at 3b and 7 (14%) at stage 4 (e-GFR > 25 mL/min). The dose of tolvaptan was adjusted based on urine osmolarity (<200 mOsm/kg in a morning urinary specimen).

At 18 months, 19, 14 and 18 patients were treated with 90/30, 60/30 and 45/15 mg/day tolvaptan, respectively. Patients were encouraged to drink water according to their thirst, but the consumed water amount had to be close to the urine volume produced and measured every month. Prior to initiation and monthly during the 18-month treatment period, we measured urine osmolality, serum creatinine, sodium, potassium, calcium, phosphorus, uric acid and 24 h urine volume in every patient. The 24 h urine volume and urine osmolality prior to and during the 18 months of tolvaptan treatment are shown in Table 5. Urine osmolality and urine volume had an inverse correlation (β = −0.017, *p* < 0.001). For each 1 liter increase in the mean urine volume, the mean urine osmolality decreased by 17 mOsm/kg.

No significant associations were found between 24 h urine volume and serum parameters (including e-GFR) measured every month during the 18 months of tolvaptan treatment, as shown in Table 6.

## 4. Discussion

In the present study, conducted in real clinical practice, we demonstrated that tolvaptan treatment prolongs the clinical course of ADPKD compared to estimations of rate of progression. Although TKV was not affected, renal function, the main clinical parameter (assessed with e-GFR) was positively influenced, showing a slower than expected e-GFR decline after 3 years of tolvaptan therapy. This favorable result was enough to also change the ESKD prediction. On the other hand, although urine volume was significantly increased, no acute kidney injury or electrolyte imbalances were observed in any of the 51 patients treated for 18 months with this drug.

The first specific treatment for ADPKD, which is the fourth-leading cause of ESKD worldwide, was an important step towards disease management. Results from Tempo 3:4 and Reprise studies, as well as their open label extensions and post hoc analyses, were all positive [2,3,8]. Prolongation by 1 year of the expected clinical course of ADPKD after just 3 years of treatment, in the present study based on our daily clinical practice, is also promising.

Our finding that TKV was not significantly influenced after 3 years of tolvaptan treatment seems questionable, because kidney volume has an established inverse relationship with renal function in ADPKD. On the other hand, TKV may not be the only parameter related to the deterioration in renal function. As a recent study showed, other imaging factors, such as the total number of cysts or the surface of the renal parenchyma that encountered renal cysts, may be more tightly associated with the e-GFR decline [9].

We found that younger patients with delayed hypertension onset and older age of ESKD in the affected parent achieved better renal function preservation with tolvaptan treatment. Hypertension is critical to the clinical course of ADPKD and an independent factor for response to tolvaptan treatment [10,11,12]. On the other hand, the age at which the affected parent reached ESKD is indicative of the genetic background [13]. The site (PKD1, PKD2 or other) and the type (truncated or not, etc.) of the mutation does not differ between parent and child. Heritability of other gene polymorphisms capable of differentiating the clinical course of the disease may also be important [14].

Factors including preserved e-GFR at treatment initiation, advanced age of the parent reaching ESKD and severe MCIC (e.g., 1E vs. 1C, representative of the cyst load corrected to the height and the age of the patient) were associated with a favorable ESKD prediction after 3 years of tolvaptan treatment. Better preserved e-GFR at treatment initiation seems to be critical, supporting the fact that tolvaptan acts more efficiently (in cellular pathways) before other processes (e.g., renal fibrosis–chronic inflammation) aggravate ADPKD progression [15,16]. The finding that patients with advanced MCIC (indicative of the rapid progression of the disease) show better response to treatment probably indicates that tolvaptan inverses the rate of cyst growth more efficiently when this is more rapid [4].

Aquaretic effects of tolvaptan are the most distressing side effects for the patient taking the drug [2,3]. The increased diuresis may also have an unfavorable effect on treatment outcome. Our data showed that, in compliant ADPKD patients, massive diuresis does not have a negative impact on renal function and electrolyte balance. In fact, drinking water approximately equal to the amount of urine volume prevents any imbalance. Our results confirm that tolvaptan increases free-water clearance (i.e., acting only on aquaporin water transport at distal nephron) [17], hence not causing any electrolyte imbalances. Furthermore, urine osmolarity is sustained at stable low levels, and this effect may also be important, assuming that vasopressin is maintained at low levels too [18].

Although this is a retrospective–observational study with a limited number of patients, we believe it is noteworthy, as it largely confirms the findings of larger prospective, double-blinded placebo-controlled studies, demonstrating significant results of tolvaptan as the first specific treatment for ADPKD in the real clinical setting. The study’s significance is further enhanced by the additional information it provides regarding the aquaretic adverse effects of tolvaptan and their potential impact on renal function and electrolyte balance.

## 5. Conclusions

In conclusion, tolvaptan treatment seems to be effective in delaying the clinical course of ADPKD. Younger patients with preserved renal function, more benign familial renal history, shorter duration of hypertension and more advanced imaging category seem to benefit more from the treatment. Aquaretic side effects of the drug do not affect renal function and electrolyte balance in compliant patients.

## Figures and Tables

**Table 1:** Demographic, clinical and laboratory characteristics of 41 ADPKD patients at baseline (i.e., before tolvaptan initiation).

	Total	Male	Female	*p*-Value
Patients, n (%)	41 (100%)	23 (56%)	18 (44%)	
Age, years, mean (SD)	42.5 (8.6)	40.7 (8.6)	44.7 (8.2)	0.14
Age at ADPKD diagnosis, years, mean (SD)	24 (9.4)	21.5 (8)	27.7 (10.3)	0.04
Presence of HTN, n (%)	39 (100%)	21 (54%)	18 (46%)	0.2
Age at HTN diagnosis, years, mean (SD)	34 (8.5)	31.5 (8)	38 (8)	0.02
Age at ESKD of the affected parent, years, mean (SD)	55 (20)	54 (9)	57 (11)	0.4
MCIC 1C, n (%)	17 (41%)	9 (39%)	8 (44%)	0.84
MCIC 1D, n (%)	18 (44%)	11 (48%)	7 (39%)	
MCIC 1E, n (%)	6 (15%)	3 (13%)	3 (17%)	
CKD stage 1, n (%)	6 (15%)	5 (22%)	1 (6%)	0.47
CKD stage 2, n (%)	17 (42%)	7 (30%)	10 (55%)	
CKD stage 3a, n (%)	5 (12%)	3 (13%)	2 (11%)	
CKD stage 3b, n (%)	10 (24%)	6 (26%)	4 (22%)	
CKD stage 4, n (%)	3 (7%)	2 (9%)	1 (6%)	
TKV (mL), median (IQR)	1703 (1531)	1933 (3370)	1599 (1298)	0.85
e-GFR, mL/min/1.73 m^2^, mean (SD)	61.8 (24.6)	63.4 (28.4)	59.9 (19.4)	0.65
ESKD prediction, years, mean (SD)	13 (7)	13.7 (8)	12.3 (5.5)	0.52

ADPKD, Autosomal-Dominant Polycystic Kidney Disease; HTN, Hypertension; ESKD: End-Stage Kidney Disease; IQR, interquartile range; MCIC, Mayo Clinic Imaging Category; TKV, Total Kidney Volume.

**Table 2:** Expected TKV, e-GFR at 3 years without treatment and initial ESKD prediction vs. measured TKV, e-GFR and ESKD prediction after 3 years of tolvaptan treatment.

	Expected without Treatment at 3 Years	Measured After 3 Years of Tolvaptan Treatment	*p*-Value
TKV (mL), median (IQR) Mean (SD)	2180 (1091) 2717 (1839)	2250 (1357) 2773 (2087)	0.48
e-GFR (mL/min/1.73 m^2^), mean (SD)	51.1 (25.2)^1^	57.3 (30)	<0.001
e-GFR (mL/min/1.73 m^2^), mean (SD)	48.8 (27.4)^2^	57.3 (30)	<0.001
ESKD prediction (years), mean (SD)	10 (7)	12 (8)	<0.001

TKV, Total Kidney Volume; ESKD, End-Stage Kidney Disease.

**Table 3:** Multivariable analysis of slower e-GFR decline after 3 years of tolvaptan treatment.

	β-Coefficient	Std. Err.	*p* > |t|	[95% Conf. Interval]
Patient’s age at treatment initiation, years	−0.9	0.29	0.006	[−1.52, −0.29]
Patient’s age at hypertension diagnosis, years	0.88	0.3	0.008	[0.25, 1.52]
Age at ESKD of the affected parent, years	0.57	0.2	0.008	[0.16, 0.98]

ESKD, End Stage Kidney Disease.

**Table 4:** Multivariable analysis of prolonged ESKD prediction after 3 years of tolvaptan treatment.

	β-Coefficient	Std. Err.	*p* > |t|	[95% Conf. Interval]
e-GFR at treatment initiation mL/min/1.73 m^2^	0.06	0.017	0.001	[0.03, 0.1]
Age at ESKD of the affected parent, years	0.13	0.05	0.013	[0.03, 0.23]
MCIC (1C—1E)	1.93	0.7	0.013	[0.46, 3.39]

ESKD, End-Stage Kidney Disease; MCIC, Mayo Clinic Imaging Category.

**Table 5:** The 24 h urine volume and urine osmolality prior to and during the 18 months of tolvaptan.

	Prior to Tolvaptan Initiation	During Tolvaptan Treatment (18 Consecutive Measurements)
24 h urine volume, mL, mean (SD)	3167 (1217)	5403 (1900)
Urine osmolality, mOsm/kg, mean (SD)	354 (200)	198 (91)

**Table 6:** Associations between serum parameters (including e-GFR) and 24 h urine volume measured monthly during the 18 months of tolvaptan treatment.

	β-Coefficient	Std. Error	*p* > |z|	[95% Conf. Interval]
e-GFR, mL/min/1.73 m^2^–Urine volume (mL)	0.00002	0.0002	0.9	[−0.0004, 0.0004]
serum sodium, mEq/L–Urine volume (mL)	0.0001	0.0001	0.15	[−0.00003, 0.0003]
serum potassium, mEq/L–Urine volume (mL)	0.0001	0.0001	0.4	[−0.0002, 0.0004]
serum calcium, mg/dL–Urine volume (mL)	−6.89 × 10^–6^	0.00002	0.65	[−0.00004, 0.00002]
serum phosphorus, mg/dL–Urine volume (mL)	0.00002	0.00002	0.4	[−0.00003, 0.0001]
serum uric acid, mg/dL–Urine volume (mL)	0.00006	0.00004	0.16	[−0.000022, 0.0001]

## Data Availability

The data presented in this study is available on request from the corresponding author. The data is not publicly available due to patient’s privacy protection.
